# *Artemisia argyi* Essential Oil Inhibits Hepatocellular Carcinoma Metastasis via Suppression of DEPDC1 Dependent Wnt/β-Catenin Signaling Pathway

**DOI:** 10.3389/fcell.2021.664791

**Published:** 2021-06-29

**Authors:** Yanli Li, Yang Tian, Wei Zhong, Ning Wang, Yafeng Wang, Yan Zhang, Zhuangli Zhang, Jianbo Li, Fang Ma, Zhihong Zhao, Youmei Peng

**Affiliations:** ^1^Henan Key Laboratory for Pharmacology of Liver Diseases, Institute of Medical and Pharmaceutical Sciences, Zhengzhou University, Zhengzhou, China; ^2^School of Pharmaceutical Sciences, Zhengzhou University, Zhengzhou, China; ^3^Department of Stomatology, People’s Hospital of Zhengzhou, Zhengzhou, China

**Keywords:** *Artemisia argyi* essential oil, hepatocellular carcinoma, metastasis, DEPDC1, Wnt/β-catenin, EMT

## Abstract

The tumor metastasis is the major hurdle for the treatment of advanced hepatocellular carcinoma (HCC), due in part to the lack of effective systemic treatments. DEPDC1, a novel oncoantigen upregulated in HCC, is thought to be a molecular-target for novel therapeutic drugs. *Artemisia argyi* is a traditional Chinese medicine with anti-inflammatory and anti-tumor activities. This study investigated the potential therapeutic benefits of *Artemisia argyi* essential oil (AAEO) in suppressing metastasis of HCC by targeting DEPDC1. Assessment of AAEO cytotoxicity was performed by MTT assay. Anti-metastatic effects of AAEO were investigated *in vitro* using wound healing and transwell assays. The HepG2 cells were transduced with lentiviral vector containing luciferase (Luc). A metastasis model of nude mice was established by tail vein injection of HepG2-Luc cells. The nude mice were treated with AAEO (57.5, 115, and 230 mg/kg) or sorafenib (40 mg/kg). Metastasis of HCC cells was monitored via *in vivo* bioluminescence imaging. After treatment for 21 days, tissues were collected for histological examination and immunohistochemistry analysis. Gene and protein levels were determined by real-time quantitative PCR and western blotting. The results revealed that AAEO significantly inhibits the migration and invasion *in vitro* in a concentration-dependent manner. *In vivo* assays further confirmed that AAEO markedly inhibits HCC metastasis into lung, brain, and femur tissues and exhibits low toxicity. Our results suggested that AAEO significantly downregulates the mRNA and protein expression of DEPDC1. Also, AAEO attenuated Wnt/β-catenin signaling through reduction of Wnt1 and β-catenin production. Moreover, AAEO prevented epithelial-mesenchymal transition (EMT) by downregulation of vimentin and upregulation of E-cadherin. Furthermore, we found that *DEPDC1* promoted HCC migration and invasion via Wnt/β-catenin signaling pathway and EMT. These results demonstrate that AAEO effectively inhibits HCC metastasis via attenuating Wnt/β-catenin signaling and inhibiting EMT by suppressing DEPDC1 expression. Thus, AAEO likely acts as a novel inhibitor of the DEPDC1 dependent Wnt/β-catenin signaling pathway.

## Introduction

Hepatocellular carcinoma (HCC) is one of the most lethal cancers and the second most common cause of cancer-related deaths globally, due in part to the lack of systemic treatment options (Patel and Sun, 2014). Several therapeutic approaches exist for patients with early or intermediate stage HCC, including surgical resection, radiofrequency ablation, and liver transplantation (Vogel and Saborowski, 2020). However, most HCC cases are diagnosed in their advanced stages. The tumor metastasis is the major hurdle for the treatment of advanced HCC, which strictly limits the treatment options (Fako et al., 2016). As a multikinase inhibitor, sorafenib is the only approved systemic therapy for advanced HCC. However, its actual benefit is relatively low (Patel and Sun, 2014). Hence, it is necessary to develop novel agents that can effectively restrain HCC metastasis.

DEPDC1, DEP (disheveled, EGL-10, pleckstrin) domain-containing 1 protein, is a novel oncoantigen upregulated in multiple types of cancers, including HCC (Kanehira et al., 2007; Qu et al., 2018; Zhao et al., 2019; Zhu et al., 2020). DEPDC1 is thought to be a novel diagnostic marker for human cancers and a molecular-target for novel therapeutic drugs or cancer peptide-vaccine. Recently, a DEPDC1-derived short peptide vaccine has demonstrated promising efficacy in preventing bladder cancer recurrence in a phase I/II clinical trial (Murahashi et al., 2016; Obara et al., 2017). DEPDC1 contains a highly conserved DEP domain, which involves in signal transduction (Daniel et al., 2006). Several studies revealed that disheveled DEP domain binding to Wnt signaling receptor is a key event in precise regulation of Wnt/β-catenin signaling (Daniele et al., 2012; Jiang et al., 2015). It has been reported that the activation of Wnt/β-catenin signaling plays a key role in hepatic oncogenesis, tumor cell proliferation and metastasis (Pez et al., 2013). Few studies have shown that DEPDC1 regulates tumor proliferation and metastasis via Wnt/β-catenin signaling pathway (Yang et al., 2014; Qu et al., 2018). Thus, the relationship between DEPDC1 and Wnt/β-catenin signaling and their contributions to HCC metastasis remain to be elucidated.

*Artemisia argyi* Lévl. et Vant. is a famous plant species in traditional Chinese medicine used to control dysmenorrhea, abdominal pain, and inflammation (Editorial Board of Chinese Pharmacopoeia, 2015). Recent studies have shown that it possesses antioxidant, anti-cancer, anti-inflammatory, immunomodulatory, as well as antimicrobial properties (Li et al., 2018; Lee et al., 2020). A wide range of phytochemicals including essential oils, flavonoids, organic acids, terpenes, polysaccharides, and coumarins have been identified in *Artemisia argyi* (Song et al., 2019). Recent studies have shown that many components isolated from *Artemisia argyi* display anti-tumor activities by inducing apoptosis and reducing angiogenesis (Michael et al., 2006; Zhang et al., 2018; Dai et al., 2020). Moreover, some phytochemicals isolated from *Artemisia argyi* inhibit metastasis in multiple types of cancers (Lee et al., 2005; Jeong et al., 2007; Tseng et al., 2020). In our previous study, a total of 69 compounds were identified in *Artemisia argyi* essential oil (AAEO), including abundant monoterpenes and their derivatives (1, 8-cineole and α-terpineol), sesquiterpenoids and a comparatively minor amount of aldehydes, ketones, phenolic and aromatic compounds (Zhao et al., 2013). Further investigation of ours showed that AAEO can significantly inhibit HCC and lung cancer growth (Ding et al., 2019). However, the investigation of AAEO against HCC migration and invasion is still lacking.

In this study, we investigated the effect of AAEO against HCC metastasis by targeting DEPDC1 for the first time. We found that AAEO treatment inhibited the migration and invasion of HepG2 and SMMC-7721 cells, and markedly inhibited HCC metastasis in a mouse xenograft model. Also, AAEO significantly suppressed DEPDC1 expression, which led to the downregulation of Wnt/β-catenin signaling and epithelial-mesenchymal transition (EMT). Therefore, AAEO likely acts as a novel inhibitor of the DEPDC1 dependent Wnt/β-catenin signaling pathway, and may provide a potential strategy for the treatment of HCC.

## Materials and Methods

### Plant Materials, Extraction of Essential Oil and Gas Chromatography Analysis

*Artemisia argyi* leaves were collected from Zhumadian city, Henan province, China and identified by Prof. Dong Chengming from Henan University of Chinese Medicine. AAEO was obtained by an optimized distillation extraction method according to the Chinese Pharmacopoeia (Zhao et al., 2014). Briefly, after soaking for 10 h, 500 g of *Artemisia argyi* leaves were added to 5 L distilled water and distilled for 6 h. Anhydrous sodium sulfate was used to dehydrate the AAEO, which was subsequently stored at −20°C. A voucher specimen of *Artemisia argyi* Lévl. et Vant. (no. ZZU-20120702003) was deposited at the herbarium of the Henan Institute of Medical and Pharmaceutical Sciences, Zhengzhou University.

**FIGURE 1:**
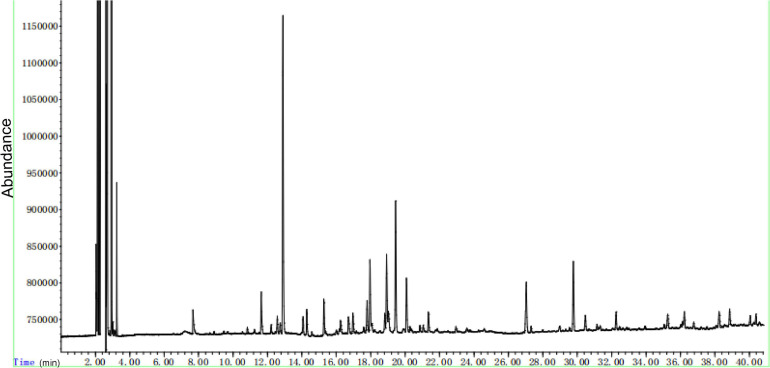
The fingerprint of AAEO performed by gas chromatography.

The fingerprint of AAEO (Figure 1) was conducted by gas chromatography (GC) method reported by our laboratory (Zhao et al., 2013; Ding et al., 2019). In brief, gas chromatographic analysis was performed on an Agilent 7890 instrument equipped with an Agilent 7683B auto-sampler. A fused silica capillary column (30 m × 0.25 mm i.d.) coated with a 0.10 μm film of crosslinked 5% phenyl methyl silicone (J&W HP-5MS) was used with helium as the carrier gas at a flow rate of 1 mL/min. The oven temperature was held for 2 min at 50°C, and then programmed at 3°C/min to 200°C, and held for 2 min, followed by 20°C/min to 280°C, and holding for 5 min. The injector temperature was 250°C. The split ratio was set to 25:1. Duplicate injections were made for each sample.

### Cell Culture and Lentiviral Transfection

HepG2 and SMMC-7721 cells were cultured in Dulbecco’s Modified Eagle Medium (DMEM) supplemented with 10% fetal bovine serum (FBS) and streptomycin at 37°C in a humidified 5% CO_2_ incubator. The HepG2 cells were transduced with a lentiviral vector containing luciferase (Luc). Subsequently, HepG2-Luc was used for establishing an HCC metastatic model in nude mice. HepG2 and SMMC-7721 cells were transduced with lentiviral vectors, resulting in *DEPDC1* overexpression (*DEPDC1*-OE) or *DEPDC1* knockdown (*DEPDC1*-KO) HCC cells, respectively. Western blotting and qRT-PCR assays were subsequently performed to confirm the expression of DEPDC1 in these cells.

### MTT Assay

HepG2, SMMC-7721, or LO2 (a normal liver cell line) cells were seeded in 96-well plate at a density of 3 × 10^3^/well. After incubation for 24 h, the cells were treated with AAEO at different concentrations (94.4, 188.8, 283.3, 377.7, 472.2, 661.1, and 944.4 μg/ml) for 72 h. Cell viability was measured by the 3-(4, 5-dimethylthiazol-2-yl)-2, 5-diphenyltetrazolium bromide (MTT) test. The CC_50_ or IC_50_ was defined as the concentration required to inhibit cell proliferation by 50%.

### Wound Healing Assay

HepG2 or SMMC-7721 cells were seeded in a six-well dish and incubated for 24 h. The monolayer was then scratched with pipette tips and washed with phosphate buffered saline (PBS). The cells were cultured in fresh medium containing AAEO at different concentrations (0, 23.6, 47.2, and 94.4 μg/mL) for 24 and 48 h. The rate of wound closure was examined and photographed by an inverted microscope (SDPTOP, China). Wound healing assay of *DEPDC1*-OE or *DEPDC1*-KO HCC cells were performed as mentioned above without AAEO treatment.

### Migration and Invasion Assays

Migration and invasion assays were performed using a transwell chamber (8 μm pores). Briefly, the chambers set into the 24-well cluster plates were coated with 20 μL matrigel and incubated at 37°C for 30 min for the cell invasion assay, while non-coated chambers were used for the cell migration assay. HepG2 and SMMC-7721 cells were resuspended in medium with different concentrations of AAEO (0, 23.6, 47.2, and 94.4 μg/mL) and seeded at a density of 2 × 10^4^ cells/well. Following 24 h incubation, the cells were fixed in methanol and stained with crystal violet. Subsequently, the number of cells that had migrated or invaded was counted using an inverted microscope (SDPTOP, China). Migration and invasion assays of *DEPDC1*-OE or *DEPDC1*-KO HCC cells were performed as mentioned above without AAEO treatment.

### Animals

Female nude mice (Balb/c nu/nu), aged between 4 and 6 weeks, were obtained from Beijing Vital River Laboratory Animal Technology Co., Ltd. (Beijing, China). The animals had free access to drinking water and feed *ad libitum*. All animals were treated in accordance with the procedures outlined in the Guide for the Care and Use of Laboratory Animals (China), and experimental procedures were approved by the Animal Ethics Committee of Zhengzhou University.

### Establishment of HCC Metastasis Model and AAEO Treatment

To establish the metastasis model, HepG2-Luc cells (2 × 10^6^) were intravenously administered via tail vein infusion. Two weeks post inoculation, 25 tumor-bearing nude mice were randomly divided into five groups (*n* = 5 per group). The tumor-bearing mice were intraperitoneally injected with AAEO (57.5, 115, and 230 mg/kg/day) or given oral dose of sorafenib (40 mg/kg/day) for 21 days. Body weight was measured every 2 days, and bioluminescence imaging was performed using IVIS Lumina III (PerkinElmer, United States). At the end of the study, all mice were sacrificed, and the tissues were collected for histopathological as well as immunohistochemical examination.

### Histopathology Examination

The tissues were fixed in 10% neutral buffered formalin, dehydrated, and embedded in paraffin. Samples were subsequently sliced at 5 μm thickness and stained with hematoxylin and eosin (H&E) for histopathology.

### Quantitative Real-Time PCR

Total RNA of cells and lung tissues was extracted using RNAiso Plus (Takara, Japan) and reverse-transcribed using the PrimeScript^TM^RT reagent kit (Takara). The primer sequences were as follows: *DEPDC1*: forward, 5′-CCATCATTGCAATAGCAGG-3′ and reverse, 5′-GAGCATACATATGTTCAAACTTC-3′; *GAPDH*: forward, 5′-CAGGAGG CATTGCTGATGAT-3′, reverse, 5′-GAAGGCTGGGGCTCATTT-3′. Quantitative real-time PCR was performed using SYBR Premix Ex Taq II (Takara) in an Applied Biosystems 12k machine (Thermo Fisher Scientific, United States). Polymerase chain reaction conditions were as follows: 95°C for 30 s for stage 1, 40 cycles at 95°C for 5 s, and 64°C for 34 s for stage 2. The relative expression was calculated using the 2^–ΔΔ*Ct*^ formula.

### Western Blotting Analysis

The cells or tissue homogenate were harvested, lysed with lysis buffer for 30 min on ice, and centrifuged at 14000 rpm for 15 min at 4°C to obtain the protein. The western blot was performed according to the manufacturer’s specification. The primary antibodies used were anti-DEPDC1 (1:1000, Abcam, United Kingdom), anti-Wnt1 (1:1000, Abcam, United Kingdom), anti-β-catenin (1:6000, Proteintech, United States), anti-vimentin (1:2000, Proteintech, United States), and anti-E-cadherin (1:4000, Proteintech, United States).

### Tissue Immunohistochemistry

Immunohistochemistry was performed as described previously. In brief, tissues were fixed with 4% paraformaldehyde, paraffin embedded, and cut into 4 μm slices. Immunohistochemical staining was conducted according to the manufacturer’s protocol. The protein expression was evaluated using Image-Pro Plus 6.0 based on the integral optical density.

### Statistical Analysis

Statistical analysis was performed using SPSS 20.0 (IBM software, Somers, NY, United States)^1^, and differences were considered statistically significant at *p* < 0.05. The differences between two groups were analyzed using a two-sided unpaired Student’s *t*-test. The differences among multiple groups were analyzed using one-way ANOVA, followed by Duncan’s multiple-range tests. All data are expressed as mean ± standard deviation (SD).

## Results

### AAEO Inhibited the Proliferation, Migration and Invasion of HCC *in vitro*

**FIGURE 2:**
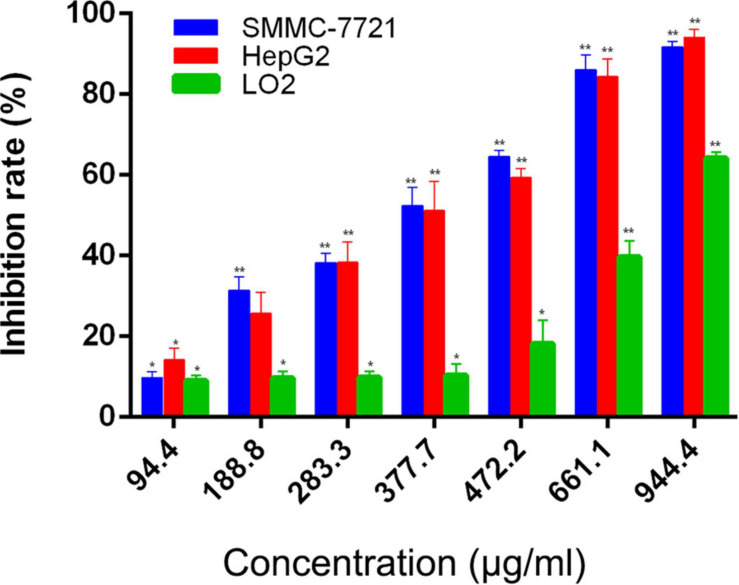
AAEO treatment inhibits cellular proliferation in SMMC-7721 and HepG2 cells. Data are expressed as the mean ± SD of three independent experiments. *p < 0.05 and **p < 0.01 vs. the control group.

**FIGURE 3:**
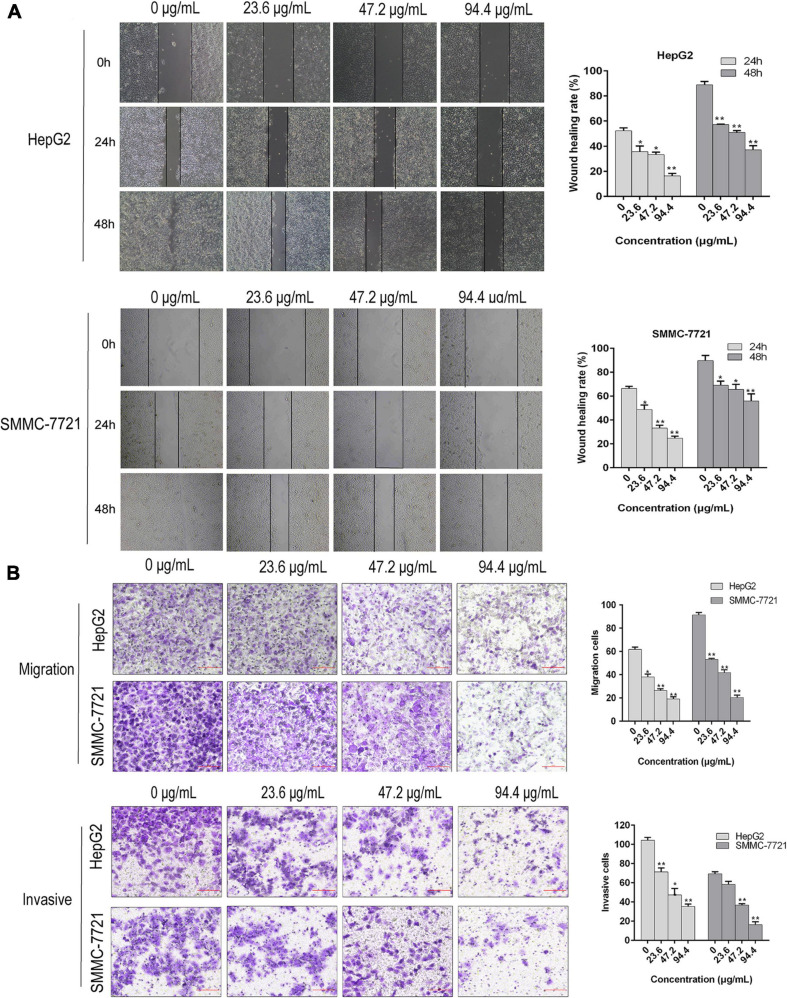
AAEO treatment suppresses the migration and invasion of HCC cells *in vitro*. **(A)** Representative images of wound healing assay (left panels) and corresponding wound healing rate (right panels) in SMMC-7721 and HepG2 cells treated with AAEO (23.6, 47.2, and 94.4 μg/mL). **(B)** Representative images of transwell migration assay (without matrigel) and invasion assays (with matrigel) in SMMC-7721 and HepG2 cells treated with AAEO for 24 h. Data are expressed as the mean ± SD of three independent experiments. *p < 0.05 and **p < 0.01 vs. the control group.

Our previous investigations have showed that AAEO prevents HCC cell proliferation *in vitro* and inhibits tumor growth in a HCC mouse xenograft model through induction of tumor cell apoptosis and blocking the cell cycle in the G2/M phase (unpublished data). In this study, AAEO exhibited low toxicity on a normal liver cell line (LO2) with a CC_50_ value of 749.6 ± 24.4 μg/mL. Moreover, it displayed significant inhibitory activity against HCC in a concentration-dependent manner, with IC_50_ values of 321.7 ± 3.8 and 322.0 ± 14.3 μg/mL for HepG2 and SMMC-7721 cells, respectively (Figure 2). To characterize the effect of AAEO on HCC migration and invasion, we chose to use ≤94.4 μM (∼IC_10_) AAEO for subsequent studies. Wound healing assay suggested that AAEO suppressed the migration of SMMC-7721 and HepG2 cells in a concentration-dependent manner. Moreover, transwell migration and invasion assays showed that both SMMC-7721 and HepG2 cells treated with AAEO displayed a marked decrease in migration and invasion in comparison to untreated cells (Figure 3). These results suggested that AAEO exerts potent inhibitory effects on the proliferation, migration and invasiveness of HCC cells.

### AAEO Suppressed the Metastasis of HCC in Nude Mice

**FIGURE 4:**
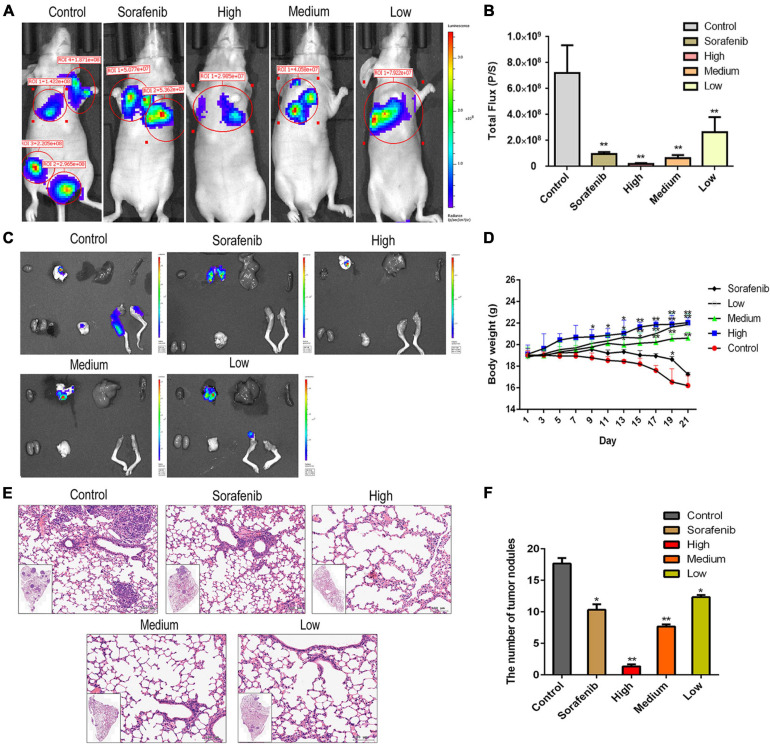
AAEO treatment inhibits the lung, brain, and femur metastases of HCC cells in nude mice. Two weeks after inoculation with HepG2-Luc cells, tumor-bearing nude mice were treated with AAEO (57.5, 115, and 230 mg/kg) or sorafenib (40 mg/kg) for 21 days. **(A)** Representative bioluminescence imaging tracer results of nude mice at the end of study. **(B)** Quantification of bioluminescence imaging signal intensities in nude mice (*n* = 5 per group). **(C)** IVIS imaging performed on nude mice tissues after dissection. **(D)** Body weight of tumor-bearing mice from different groups during treatment period. **(E)** Representative H&E-stained histologic images of tumor sections. **(F)** The number of lung metastatic nodules. *p < 0.05 and **p < 0.01 vs. the control group.

To determine the anti-metastatic effects of AAEO *in vivo*, a metastasis animal model was established in nude mice. Two weeks post inoculation, lung metastasis was observed by *in vivo* bioluminescence imaging. On day 21, bioluminescence imaging results showed that the tumor had metastasized into the lung, brain, and femur tissues in the control group (Figures 4A–C). After AAEO treatment for 21 days, tumor metastasis significantly decreased in a dose-dependent manner. The inhibitory effect of AAEO (115 mg/kg) was similar to that of the sorafenib group (40 mg/kg), and AAEO at a dose of 230 mg/kg showed a higher anti-metastasis effect than the sorafenib group (Figures 4A–C). Additionally, body weight was monitored throughout the experiment and found to decrease in untreated mice, along with tumor metastasis and progression. Although sorafenib treatment inhibited tumor metastasis, sorafenib-treated mice exhibited obvious weight loss (Figure 4D), which may be caused by drug systemic toxicity (Llovet et al., 2008). Interestingly, AAEO significantly suppressed tumor metastasis and no body weight loss was observed over the course of therapy, suggesting potent activity and low toxicity. Moreover, H&E staining results indicated that the number and size of pulmonary metastasis nodules were markedly reduced in mice treated with AAEO (115 and 230 mg/kg) and lower than those in the sorafenib group (Figures 4E,F). These results suggested that AAEO exhibits potent anti-HCC metastasis activity with low toxicity.

### AAEO Suppressed DEPDC1 Expression

**FIGURE 5:**
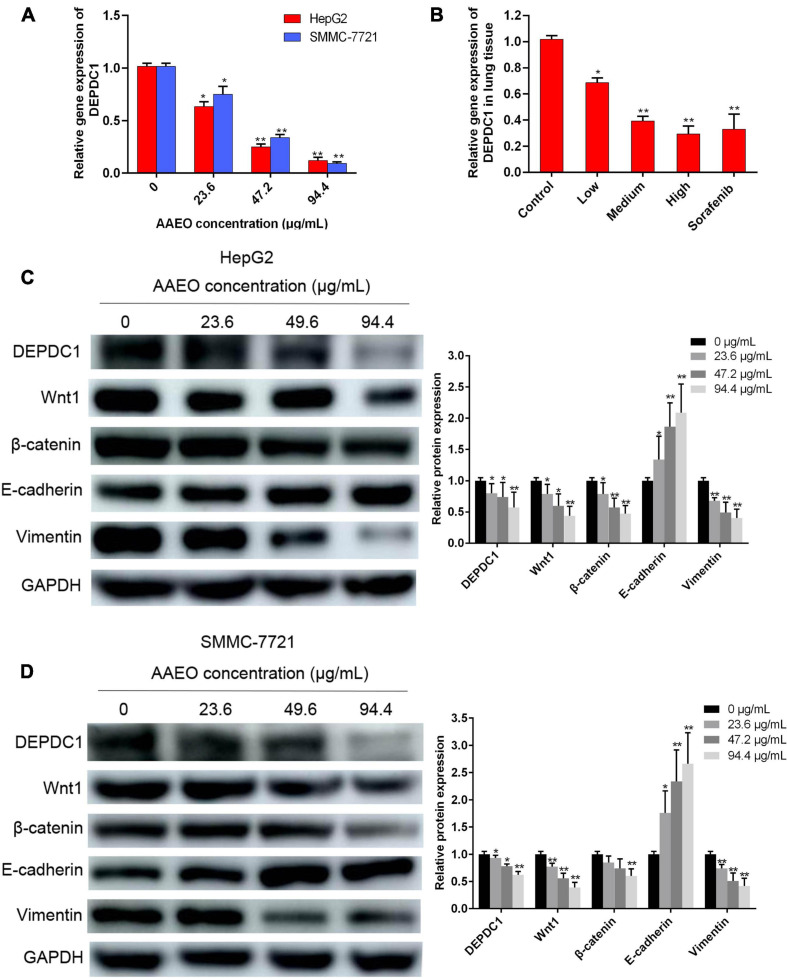
AAEO treatment suppresses DEPDC1 expression, attenuates Wnt/β-catenin signaling, and prevents EMT. **(A)** Fold changes in *DEPDC1* expression in HepG2 and SMCC-7721 cells after AAEO treatment for 72 h. **(B)** Fold changes in *DEPDC1* expression in lung metastasis tissue from nude mice after AAEO treatment for 21 days. **(C,D)** DEPDC1, Wnt1, β-catenin, and EMT related protein expression in HepG2 cells **(C)** and SMMC-7721 cells **(D)** assessed by western blotting after AAEO treatment for 72 h. Data are expressed as the mean ± SD of three independent experiments. *p < 0.05 and **p < 0.01 vs. the control group.

Increasing evidences suggest that DEPDC1 appears to be involved in the regulation of carcinogenesis and progression of HCC and represents a potential therapeutic and preventive target of HCC (Qu et al., 2018; Amisaki et al., 2019). In this study, AAEO effectively inhibited HCC metastasis *in vitro* and *in vivo.* To explore the possible involvement of DEPDC1, we investigated the effect of AAEO on DEPDC1 expression using SMMC-7721 and HepG2 cells. After treatment with AAEO for 72 h, both SMMC-7721 and HepG2 cells displayed a marked decrease in migration and invasion, while both mRNA and protein expression levels of DEPDC1 were obviously decreased in a dose-dependent manner (Figures 5A,C,D). Furthermore, the mRNA expression levels of *DEPDC1* in lung metastasis tissues were determined in nude mice bearing HCC tumors. Compared to the control group, the number and size of pulmonary metastasis nodules were markedly reduced in the AAEO group, while the mRNA levels of *DEPDC1* were significantly reduced in a dose-dependent manner (Figure 5B). These results supported that DEPDC1 may be a potential molecular target of HCC metastasis (Qu et al., 2018; Amisaki et al., 2019).

### AAEO Suppressed Wnt/β-Catenin Signaling and EMT

**FIGURE 6:**
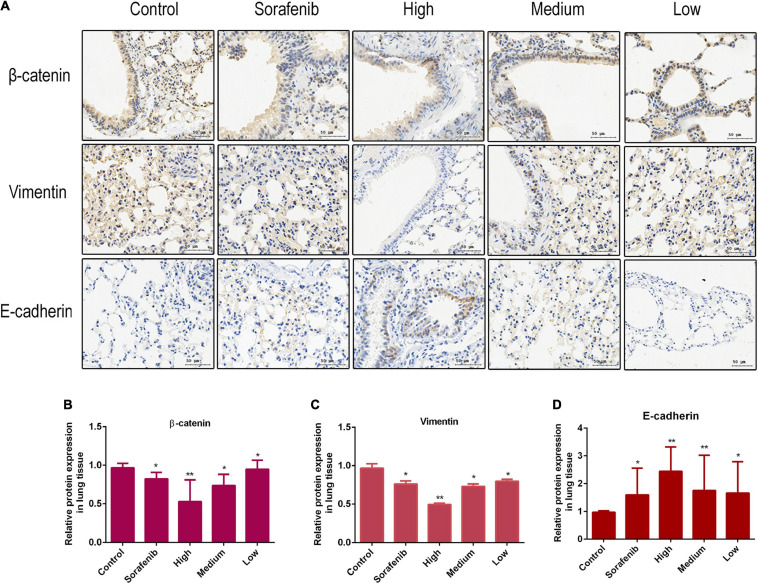
AAEO treatment suppresses Wnt/β-catenin signaling and EMT in nude mice bearing tumors. **(A)** The expression of β-catenin, E-cadherin, and vimentin examined by immunohistochemistry. **(B–D)** Fold changes in β-catenin and EMT related protein expression in lung metastasis tissue. Data are expressed as the mean ± SD of three independent experiments. *p < 0.05 and **p < 0.01 vs. the control group.

The Wnt/β-catenin pathway is broadly involved in the process of promoting tumorigenicity, cell stemness and EMT induction in multiple types of cancers, including HCC (Mosimann et al., 2009; Craene and Berx, 2013). To determine whether Wnt/β-catenin pathway was prevented by AAEO, the protein levels of Wnt1 and a downstream node in the pathway, β-catenin, were determined in AAEO-treated SMMC-7721 and HepG2 cells. A significant reduction in Wnt1 and β-catenin was observed in AAEO-treated SMMC-7721 and HepG2 cells in a concentration-dependent manner (Figures 5C,D). Furthermore, the β-catenin protein levels in lung metastasis tissues were determined in nude mice bearing HCC tumors. The results showed that AAEO treatment blocked the β-catenin production in lung metastasis tissues in a dose-dependent manner (Figures 6A,B).

Moreover, we found that AAEO treatment, in a dose-dependent manner, downregulated the expression of mesenchymal marker (vimentin), whereas it upregulated the expression of epithelial marker (E-cadherin) in SMMC-7721 and HepG2 cells (Figures 5C,D). Consistent with the *in vitro* results, AAEO significantly regulated the protein expression of vimentin and E-cadherin in lung metastasis tissues in mouse xenograft model (Figures 6A,C,D). All these results indicated that AAEO suppresses Wnt/β-catenin signaling and EMT *in vitro* and *in vivo*.

### AAEO Inhibits HCC Metastasis via Suppression of DEPDC1 Dependent Wnt/β-Catenin Signaling Pathway

**FIGURE 7:**
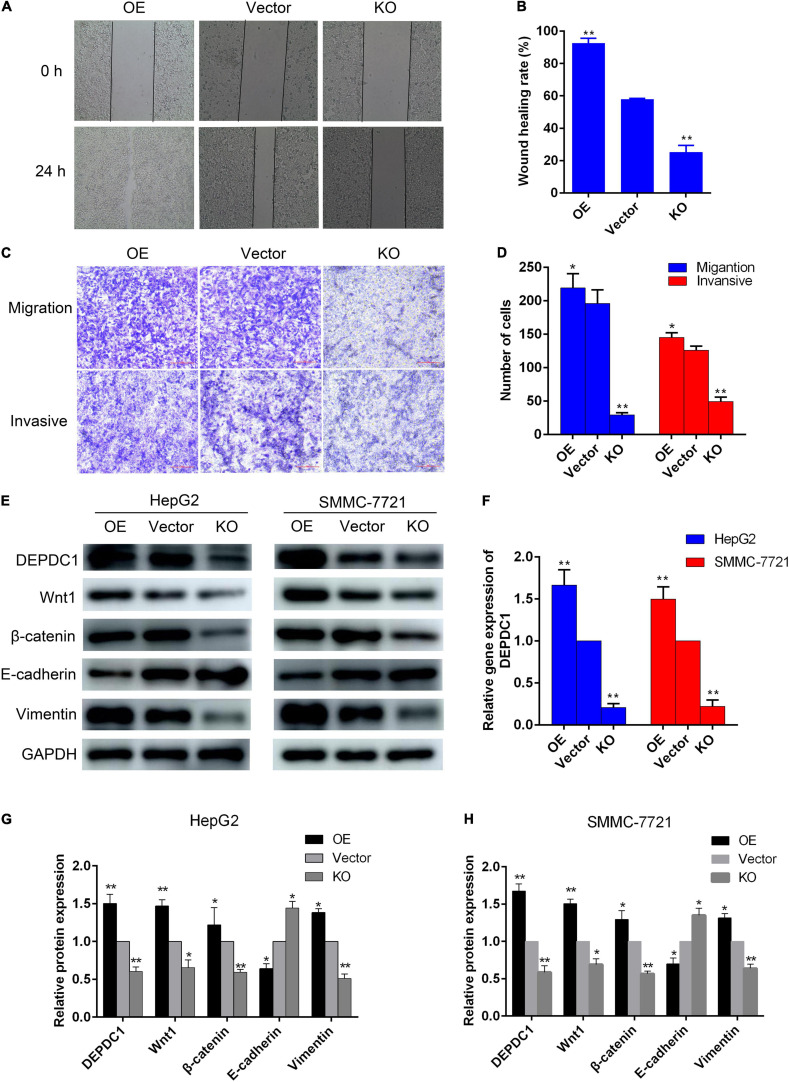
DEPDC1 promotes HCC migration and invasion via Wnt/β-catenin signaling pathway and EMT. **(A)** Representative images of wound healing assay. **(B)** Wound healing rate. **(C)** Representative images of transwell migration assay (without matrigel) and invasion assays (with matrigel). **(D)** Migration and invasion cell number. **(E)** Western blot analysis of DEPDC1, Wnt/β-catenin, and EMT related proteins in *DEPDC1*-OE and *DEPDC1*-KO HCC cells. **(F)** Fold changes in DEPDC1 gene expression. **(G,H)** Fold changes in DEPDC1, Wnt/β-catenin, and EMT related protein expression. Data are expressed as the mean ± SD of three independent experiments. *p < 0.05 and **p < 0.01 vs. the vector group.

Evidence has indicated that DEPDC1 promotes Wnt/β-catenin signaling pathway in gene set enrichment analysis of RNA sequence data and validated in mRNA level in SMMC-7721 cells (Wang et al., 2020). To further confirm the relationship between DEPDC1 and Wnt/β-catenin signaling pathway, HepG2 and SMMC-7721 cells were transduced with lentiviral vectors. The mRNA and protein levels of DEPDC1 were significantly reduced in *DEPDC1*-KO HCC cells, while the mRNA and protein levels were increased in *DEPDC1*-OE HCC cells (Figures 7E,F). *DEPDC1*-KO led to significant decrease in the migration and invasion, while *DEPDC1*-OE exhibited increased migration and invasion (Figures 7A–D). Furthermore, the protein levels of Wnt1 and β-catenin were substantially reduced in *DEPDC1*-KO HCC cells. In contrast, the protein levels of Wnt1 and β-catenin were increased in *DEPDC1*-OE HCC cells (Figures 7E,G,H). These results indicated that *DEPDC1* promoted HCC migration and invasion via Wnt/β-catenin signaling pathway. Moreover, *DEPDC1*-KO inhibited EMT by downregulation of vimentin and upregulation of E-cadherin expression. In contrast, EMT was increased in *DEPDC1*-OE HCC cells (Figures 7E,G,H). These results demonstrated that *DEPDC1* promoted HCC migration and invasion via Wnt/β-catenin signaling and EMT. In the present study, AAEO treatment significantly downregulated DEPDC1 expression, attenuated Wnt/β-catenin signaling and inhibited EMT both *in vitro* and *in vivo*. Taken together, AAEO inhibits HCC metastasis via suppression of DEPDC1 dependent Wnt/β-catenin signaling pathway.

## Discussion

The tumor metastasis is the major hurdle for the treatment of advanced HCC, due in part to the lack of effective systemic treatments. Currently, the multi-kinase inhibitor sorafenib is the only systemic chemotherapeutic drug approved for the treatment of HCC. However, clinical data have indicated that sorafenib prolonged the median survival by approximately 3 months in patients with advanced stage HCC and acquired resistance to sorafenib over the course of therapy is likely (Llovet et al., 2008; Leung et al., 2020). Additionally, HCC is intrinsically resistant to a number of cytotoxic agents. Natural products are generally considered as safe supplier for biomedical applications due to their low toxicity (Kim et al., 2020). In cancer therapy, naturally derived agents represent the largest portion of approved anti-tumor drugs (Newman and Cragg, 2016). *Artemisia argyi* is a traditional Chinese medicine with multiple biological effects. Recent studies have shown that it possesses anti-cancer activities in multiple types of cancers (Michael et al., 2006; Li et al., 2018; Zhang et al., 2018; Dai et al., 2020). Our previous study suggested that AAEO displays anti-hepatitis B virus activity (Zhao et al., 2016). Also, we found that AAEO prevented HCC cell proliferation *in vitro* and inhibited tumor growth in a HCC mouse xenograft model through induction of apoptosis and blocking the cell cycle in the G2/M phase (unpublished data). In this study, we further investigated the anti-metastatic activity of AAEO on HCC.

Our results demonstrated that AAEO can significantly inhibit the migration and invasion of SMMC-7721 and HepG2 cells in a concentration-dependent manner. Further *in vivo* investigations showed that it exhibits potent anti-HCC metastasis activity in a dose-dependent manner. The inhibitory effect in the AAEO (115 mg/kg) group was similar to that in the sorafenib group (40 mg/kg), and the AAEO (230 mg/kg) group showed a higher anti-metastasis effect than the sorafenib group. Clinical data have indicated that the benefit of sorafenib is relatively low for patients with the worst prognosis, such as those with macroscopic vascular invasion or extrahepatic spread (Llovet et al., 2008). Moreover, diarrhea, weight loss, hand-foot skin reaction, and hypophosphatemia occurred at a high frequency for patients receiving sorafenib. In the present study, although sorafenib inhibited tumor metastasis, sorafenib-treated mice exhibited obvious weight loss, which may be caused in part by drug systemic toxicity. Interestingly, AAEO significantly suppressed tumor metastasis and no body weight loss was observed over the course of therapy, suggesting potent activity and low toxicity.

DEPDC1 is a highly conserved protein across many species, from *Caenorhabditis elegans* to mammals (Sendoel et al., 2014). It is involved in signal transduction and regulates several cellular functions, including a large number of signaling proteins (D’ Andrea et al., 2014; Dibble and Cantley, 2015). *DEPDC1* was first identified as a novel gene in bladder cancer, wherein it plays an essential role in the growth of bladder cancer cells (Harada et al., 2010). In recent years, several reports have also shown that *DEPDC1* is aberrantly upregulated in various tumors and is involved in the occurrence and development of HCC (Amisaki et al., 2019; Guo et al., 2019), breast cancer (Zhao et al., 2019), lung cancer, colorectal cancer (Wang et al., 2020), and glioblastoma (Kikuchi et al., 2017). A DEPDC1-derived short peptide vaccine has demonstrated promising efficacy in preventing bladder cancer recurrence in a phase I/II clinical trial (Murahashi et al., 2016; Obara et al., 2017). However, there is no existing drug targeted to DEPDC1 for the treatment and prevention of tumor metastasis. In this study, we found that AAEO significantly inhibited the migration and invasion of HepG2 and SMMC-7721 cells by downregulating the expression of *DEPDC1*. Moreover, *in vivo* metastasis assays confirmed that AAEO markedly inhibited the expression of *DEPDC1* and reduced lung metastasis of HCC. The results from our study supported that DEPDC1 is likely to represent a potential therapeutic target of HCC (Amisaki et al., 2019; Guo et al., 2019).

The canonical Wnt pathway is one of the most frequently deregulated pathways in HCC. Evidence suggests that the canonical Wnt signaling pathway plays essential roles in cell cycle, trafficking, signaling, and migration in HCC (Zhi et al., 2015). During tumor pathogenesis, Wnt signaling collaborate to induce activation of the EMT program, which enables carcinoma cells to acquire cellular traits associated with high-grade malignancy, including the ability to complete various steps of the metastatic cascade (Scheel et al., 2011). A recent study has suggested that DEPDC1 may regulate tumor proliferation and metastasis via Wnt/β-catenin signaling pathway in HCC (Qu et al., 2018). However, the result was just based on gene set enrichment analysis of RNA sequence data and the genes change in mRNA level in SMMC-7721 cells. Thus, the relationship among DEPDC1, Wnt/β-catenin signaling and EMT remains to be elucidated. In this study, we found that *DEPDC1*-KO reduced migration and invasion ability of HCC cells by downregulation of Wnt/β-catenin signaling and inhibition of EMT. In contrast, *DEPDC1*-OE promoted migration and invasion via upregulation of Wnt/β-catenin signaling and induction of EMT in HCC cells. Moreover, AAEO inhibited HCC metastasis via suppression of DEPDC1 dependent Wnt/β-catenin signaling pathway. These results supported DEPDC1 dependent Wnt/β-catenin signaling pathway is likely to play an essential role in HCC metastasis and represent a promising therapeutic target.

Previous reports have demonstrated that DEPDC1 promotes cell proliferation, invasion, and angiogenesis in numerous cancers regulated by multiple signaling pathways. For example, DEPDC1 has been shown to activate NF-κB and E2F signaling pathways to accelerate cell cycle progression, and to promote the K-RAS and Wnt/β-catenin signaling pathways to drive tumor cell proliferation and metastasis (Feng et al., 2017; Huang et al., 2017; Qu et al., 2018). Also, DEPDC1 has been shown to drive HCC cell proliferation, invasion, and angiogenesis by regulating CCL20/CCR6 signaling pathway (Guo et al., 2019). These mechanisms may have importance in HCC proliferation and metastasis. Thus, continued examination of AAEO against HCC proliferation and metastasis warrants further investigation.

## Data Availability Statement

The original contributions presented in the study are included in the article/supplementary material, further inquiries can be directed to the corresponding authors.

## Ethics Statement

The animal study was reviewed and approved by the Animal Ethics Committee of Henan Institute of Medical and Pharmaceutical Sciences. Written informed consent was obtained from the owners for the participation of their animals in this study.

## Author Contributions

YP and ZhZ conceived and designed the study. YL, YT, and NW performed the experiments. WZ, YW, YZ, ZlZ, JL, and FM analyzed the data. All authors read and approved the final manuscript.

## Conflict of Interest

The authors declare that the research was conducted in the absence of any commercial or financial relationships that could be construed as a potential conflict of interest.

## Footnotes

1 www.ibm.com

## Abbreviations

AAEO: *Artemisia argyi* essential oil
DMEM: Dulbecco’s Modified Eagle Medium
EMT: epithelial-mesenchymal transition
HCC: hepatocellular carcinoma
H&E: hematoxylin and eosin staining
MTT: 3-(4, 5-dimethylthiazol-2-yl)-2, 5-diphenyltetrazolium bromide
PBS: phosphate buffered saline
RT-qPCR: real-time quantitative PCR.
